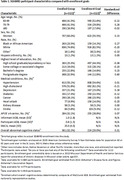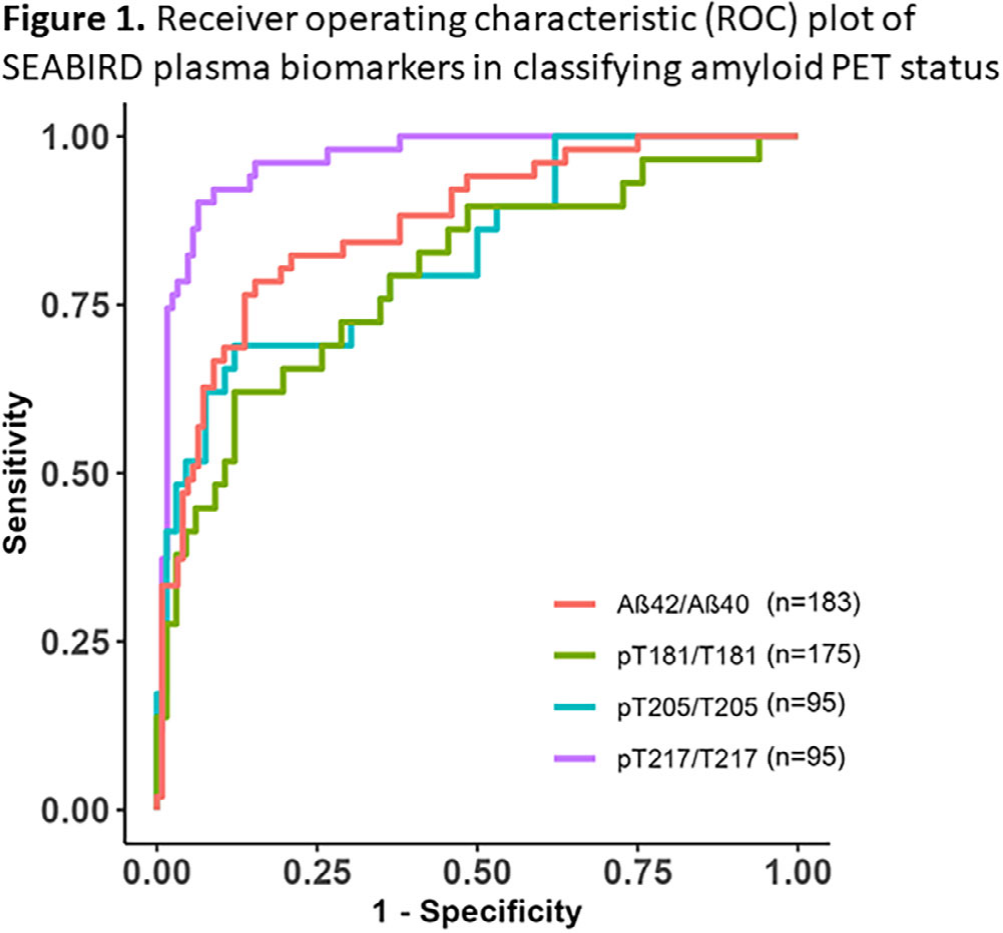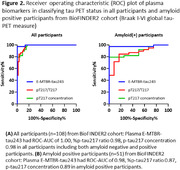# Alzheimer’s disease blood tests of amyloid‐beta 42/40, %p‐tau217, 181, and 205 ratios and MTBR‐243 in real‐world populations: Results from SEABIRD and BioFINDER2

**DOI:** 10.1002/alz.088405

**Published:** 2025-01-09

**Authors:** Randall J. Bateman, Melody Li, Nicolas R. Barthélemy, James G. Bollinger, Yingxin He, Kanta Horie, Rama Koppisetti, Vitaliy Ovod, Suzanne E. Schindler, Siobhan Sutcliffe, Ganesh M. Babulal, Tammie L.S. Benzinger, Eric J. Lenze, Brian A. Gordon, Oskar Hansson, Yan Li

**Affiliations:** ^1^ Washington University School of Medicine, St. Louis, MO USA; ^2^ The Tracy Family SILQ Center, Washington University School of Medicine, St. Louis, MO USA; ^3^ The Tracy Family SILQ Center, St. Louis, MO USA; ^4^ Washington University in St. Louis School of Medicine, St. Louis, MO USA; ^5^ Knight Alzheimer Disease Research Center, St. Louis, MO USA; ^6^ Mallinckrodt Institute of Radiology, Washington University in St. Louis, St. Louis, MO USA; ^7^ Clinical Memory Research Unit, Department of Clinical Sciences Malmö, Faculty of Medicine, Lund University, Lund Sweden

## Abstract

**Background:**

Alzheimer’s disease (AD) blood tests that can identify and quantify amyloid plaques, tau tangles, and cognitive and clinical decline are needed in clinical practice, including primary care. However, these tests require validation in diverse groups and real‐world settings. The Study to Evaluate Amyloid in Blood and Imaging Related to Dementia (SEABIRD) enrolled 1,122 participants to determine the accuracy and validity of AD blood biomarkers, including amyloid‐β (Aβ) and phosphorylated tau (p‐tau) tests, in a diverse, community‐based sample of older adults compared with amyloid PET and cognitive and clinical measures to determine the impact of key factors (age, race, education, cognition, APOE genotype, and medical conditions).

**Method:**

SEABIRD measured blood plasma Aβ42/40, %p‐tau217, %p‐tau181 and %p‐tau205 ratios by immunoprecipitation‐mass spectrometry. Measures of cognitive and clinical impairment included the AD8® dementia screen, Montreal Cognitive Assessment (MoCA), and the Clinical Dementia Rating (CDR®). 26% of participants completed amyloid PET for comparison to the blood tests. The novel plasma endogenous microtubule binding region of tau with 243 residue (E‐MTBR‐tau243) was measured in a separate cohort, the Swedish BioFINDER2 study, and compared to tau PET for tau tangle assessment.

**Result:**

Of the 1,122 participants enrolled in SEABIRD, 23.5% self‐identified as Black or African American and the percentage of APOE ε4 carriers was 32.1%, as expected for a general population (Table 1). The classification accuracy (ROC AUC) of plasma measures for amyloid PET status was evaluated in a subset of participants: Aβ42/40 0.88, %p‐tau217 ratio 0.96, %p‐tau181 ratio 0.79, and %p‐tau205 ratio 0.83 (Figure 1). The classification accuracy for tau PET status was evaluated in amyloid PET positive participants in BioFINDER2: plasma E‐MTBR‐tau243 0.98, %p‐tau217 ratio 0.87, p‐tau217 concentration 0.89 (Figure 2).

**Conclusion:**

In a diverse population with high comorbid disease burden, AD blood tests using ratios had similar accuracies to those reported in AD research cohorts. Our findings support the clinical use of these tests for detection of both amyloid and tau AD pathologies in the general population. Further, screening with AD blood tests can accelerate enrollment of more diverse cohorts into clinical trials, enabling treatments to demonstrate effectiveness in representative populations.